# The Clinical Significance of Uric Acid Deposits

**Published:** 1887-02

**Authors:** F. R. Campbell


### Translations.

### *THE CLINICAL SIGNIFICANCE OF URIC ACID DEPOSITS.*

Translated from *Nordiskt Medicinskt Arkiv.* by F. R. CAMPBELL, M. D.

Prof. Johannes Mygge, of Christiana, formerly Chief of the Medical Clinic of Copenhagen, observing that abundant and persistent uric acid deposits often coincide or alternate with albuminuria, has undertaken to make a thorough investigation of this subject. In order to ascertain the frequency of these deposits and the special conditions in which they are present he made daily examinations of the urine of 272 patients in his wards with the following results:

1. Of 3,287 specimens of urine examined, 2,786 coming from 127 patients presented no traces of uric acid deposits. But 501 specimens coming from 105 patients contained such deposits. Among these latter only 262 coming from fifty-nine showed abundant deposits; in the remainder, the quantity was small and often combined with urates. In forty-three of the fifty-nine patients whose urine gave abundant deposits, these were observed only for short periods, but in the remaining sixteen the deposits were more persistent, being observed for a week or more.

2. The deposits under consideration were observed most frequently in certain diseases, among which the following may be mentioned as the most important: They were detected in three of sixteen cases of acute articular rheumatism, and in two of eleven cases of chronic rheumatism. This same rule may be applied to the transitory deposits which are found very frequently in acute pneumonia—eleven cases in twenty-five.

3. In twenty-seven of the fifty-nine cases the author observed albuminuria coincident with the uric acid deposits and in twenty-two other cases a doubtful trace of albumen is mentioned. It is quite probable that the association of albuminuria with uric acid deposits might have been detected in other cases if an examination for albumen had been made in every case.

4. Deposits (transitory) of uric acid often appear on the cessation of attacks of acute albuminuria, a fact which has already been pointed out by Dickinson.

5. The probability of a relation between uric acid deposits and kidney lesions is indicated by the fact that seventeen of twenty-five cases in which a microscopic examination of the urine was made, morphological elements, coming from the kidneys, were detected. In fourteen cases both tube casts and renal epithelium were observed; in three cases casts only were found.

It therefore follows that there is a very frequent, if not a constant, association of renal lesions with persistent uric acid deposits, and we may also conclude that transitory deposits indicate a functional derangment of the kidneys.

The explanation of these relations is at present impossible. In some cases we may suppose that the saturation of the urine with uric acid and the consequent excessive acidity inflame the renal epithelium, and produce lesions of the kidneys. In other cases, the renal lesions undoubtedly precede the deposits of uric acid, and here we may perhaps accept the hypothesis recently advanced by Eschbach, who claims that the precipitation of the uric acid is due to the morphological elements contained in the urine. These latter deposits are only found after the urine has been passed several hours, and are of great importance not only in diagnosis but also in determining the treatment. In some cases, the uric acid was detected immediately after the urine was passed, and it is quite probable that the precipitation takes place in the urinary organs. In comparing the patients of the first group, those with uric acid deposits persisting for a week or a month, the author found that of thirty-two well-marked cases nine had infallible signs of renal disease, ten were treated for acute rheumatism, and three each for pneumonia empyæma and dysentery. Nine of the thirty-two cases were fatal. In the case of one an autopsy could not be obtained, but he presented the clinical history of nephritis. Seven of the remaining eight on post mortem examination revealed the presence of renal disease. Four had chronic nephritis and the other three had tubercular kidney, gouty kidney and cancer. In the eighth case no signs of renal disease were evident to the eye, and a microscopic examination was not made. With regard to the clinical history of these thirty-two cases, twenty-one had albuminuria, and in the twenty-five cases in which the urine was examined microscopically the author found tube casts and epithelium without exception.

Uric acid deposits are also connected with desqumation of the vesical epithelium and with certain forms of incontinence of urine. The author has not yet completed his researches regarding these latter questions.

